# Multifunctional Metallothioneins as a Target for Neuroprotection in Parkinson’s Disease

**DOI:** 10.3390/antiox12040894

**Published:** 2023-04-06

**Authors:** Ikuko Miyazaki, Masato Asanuma

**Affiliations:** Department of Medical Neurobiology, Okayama University Graduate School of Medicine, Dentistry and Pharmaceutical Sciences, Okayama 700-8558, Japan; asachan@cc.okayama-u.ac.jp

**Keywords:** metallothionein, Parkinson’s disease, neuroprotection, antioxidant, metal, synuclein, astrocyte, enteric glial cell

## Abstract

Parkinson’s disease (PD) is characterized by motor symptoms based on a loss of nigrostriatal dopaminergic neurons and by non-motor symptoms which precede motor symptoms. Neurodegeneration accompanied by an accumulation of α-synuclein is thought to propagate from the enteric nervous system to the central nervous system. The pathogenesis in sporadic PD remains unknown. However, many reports indicate various etiological factors, such as oxidative stress, inflammation, α-synuclein toxicity and mitochondrial impairment, drive neurodegeneration. Exposure to heavy metals contributes to these etiopathogenesis and increases the risk of developing PD. Metallothioneins (MTs) are cysteine-rich metal-binding proteins; MTs chelate metals and inhibit metal-induced oxidative stress, inflammation and mitochondrial dysfunction. In addition, MTs possess antioxidative properties by scavenging free radicals and exert anti-inflammatory effects by suppression of microglial activation. Furthermore, MTs recently received attention as a potential target for attenuating metal-induced α-synuclein aggregation. In this article, we summarize MTs expression in the central and enteric nervous system, and review protective functions of MTs against etiopathogenesis in PD. We also discuss neuroprotective strategies for the prevention of central dopaminergic and enteric neurodegeneration by targeting MTs. This review highlights multifunctional MTs as a target for the development of disease-modifying drugs for PD.

## 1. Introduction

Parkinson’s disease (PD) is a progressive neurodegenerative disease with motor symptoms, such as akinesia/bradykinesia, tremor, rigidity and postural instability, due to a loss of nigrostriatal dopaminergic neurons. PD patients also exhibit non-motor symptoms, such as hyposmia, constipation, and REM sleep behavior disorder (RBD), which precede motor symptoms [1]. Pathologically, neurodegeneration accompanied by an accumulation of α-synuclein, Lewy bodies and neurites is observed in the central and peripheral nervous systems of sporadic PD patients [2,3]. Currently, it is hypothesized that PD pathology propagates from the enteric nervous system (ENS) to the central nervous system (CNS) via the vagal nerve [4]. Although the pathogenic mechanism of PD has not been elucidated, various etiological factors, such as oxidative stress, inflammation, α-synuclein toxicity and mitochondrial impairment, are known to drive neurodegeneration. Environmental exposure to metals, including Fe, Cu, Zn and Mn, is thought to increase the risk of developing PD [5]. Epidemiological studies demonstrated an association of metal exposure with an increased probability of PD onset [6]. In addition, abnormal high contents of Fe and Zn in the substantia nigra (SN) were detected in post-mortem brains of PD patients [7,8].

Metallothioneins (MTs) are cysteine-rich metal-binding proteins; MTs bind metal ions (metals), such as Zn, Cu and Cd, to function in metal homeostasis and detoxification- [9]. In addition, MTs possess antioxidative, antiapoptotic and anti-inflammatory properties [10]. Because MTs can bind Cu with a high affinity, MTs can be a potential target for attenuating Cu-induced α-synuclein aggregation [11]. Therefore, MTs can play an important role in the biological function and neuroprotection against PD. Our previous studies demonstrated that upregulation of MTs could protect not only dopaminergic neurons in the SN but also enteric neurons in the ileum in parkinsonian models [12,13,14,15,16]. Moreover, several studies reported upregulation of MT expression in the brains of PD patients [17,18,19].

In this review, we review and discuss the potential protective function of MTs against etiopathogenesis in PD. We also review therapeutic strategies to prevent neurodegeneration not only in the brain but also intestinal myenteric plexus in PD by targeting MTs. Understanding the multiple protective functions of MTs will aid in the development of a disease-modifying drug for PD in future studies.

## 2. Gene Expression and Induction of MTs by Stimulation

### 2.1. Expression and Induction of MTs in the CNS

In mammalian cells, the *MT* family comprises four major genes: *MT1A*, *MT2A*, *MT3* and *MT4*. The two abundant proteins, MT1A and MT2A, share very high sequence homology and have similar expression profiles and function; therefore, they are usually considered as a single form (denoted MT1A/2A or MT1/2 in this review). MT1/2 are expressed in most organs including the brain. MT1A/2A are mainly produced in astrocytes but not neurons [20,21]. MT3, also known as growth inhibitory factor (GIF), is a predominantly brain-specific isoform [22]. MT3 is expressed primarily in neurons, especially in the Zn-containing neurons of the hippocampus, amygdala and cerebral cortex [23]. MT4 is thought to be restricted to the squamous cell epithelium. *MT1A*, *MT2A* and *MT3* mRNA are expressed constitutively in the CNS, e.g., in the olfactory bulb, cortex, caudate, hippocampus, thalamus, cerebellum and brain stem, and the quantitative order of content is MT1A > MT3 > MT2A. The olfactory bulb contains the highest mRNA expression of all types of MTs [24], implying their barrier function in the region.

MT expression is induced in response to various stimulations such as metal exposure, oxidative stress and inflammatory cytokines. In particular, MT1/2 are highly inducible by exposure to many heavy metals, including Zn, Cd and Cu. Metal regulation of *MTs’* gene expression has been well documented [25]. Induction of the *MT* genes by metals is promoted by metal-responsive element (MRE)-binding transcription factor 1 (MTF-1) [26]. MTF-1 is a multiple Zn finger protein [27]. When Zn binds to MTF-1, the transcriptional factor is translocated into the nucleus to bind MRE in the promoter region of *MT* genes, followed by induction of *MT* expression. The Zn, Cd and bismuth (Bi) activate the promoter of the *MT* genes via MRE [28], but only Zn is specific to MTF-1 [26].

MT1/2 are phase II antioxidant proteins, which are induced by oxidative stress via the Kelch-like ECH-associated protein 1 (Keap1)-nuclear factor erythroid 2-related factor 2 (Nrf2) pathway [29]. As mentioned above, astrocytes express and secrete MT1A/2A in response to oxidative stress and extracellular MTs protect surrounding neurons from oxidative stress. Our previous studies demonstrated that astrocytes produced MT1/2 by excess dopamine (DA) exposure as oxidative stress, and that extracellular MTs protected dopaminergic neurons [21]; excess DA exposure induced Nrf2 nuclear translocation and the nuclear Nrf2 could bind to antioxidant response element (ARE) in the *MT1* gene and promote *MT* expression [21]. Chung et al. also reported that MT1/2 expression is specifically upregulated in astrocytes in response to neuronal injury [30].

Acute-phase inflammatory response also induces *MTs’* expression. It is reported that bacterial endotoxin-lipopolysaccharide (LPS) and inflammatory cytokines, including interleukin (IL)-1, IL-6 and tumor necrosis factor-α (TNF-α), increase *MTs’* expression in the brain [31]. The molecular mechanism of induction of MTs by acute inflammation is not fully understood; however, intracellular Zn signaling or activation of signal transducers and activators of the transcription (STAT)-signaling pathway could be involved in inflammatory response-induced MTs’ upregulation [32].

MT1A/2A are sensitively induced in response to various stimuli in astrocytes and released extracellularly. Extracellular MT1A/2A interact with cell surface receptors of the lipoprotein receptor-related protein family, including lipoprotein receptor-related protein 1 (LRP1) and megalin, and then MTs are taken up by neurons [30]. Therefore, MT1A/2A function not only in the extracellular space but also in neurons.

### 2.2. Expression and Induction of MTs in the ENS

MT1/2 are also expressed in the ENS. Our previous studies demonstrated that GFAP-positive enteric glia in the ENS, which possess an antioxidative property similar to that of astrocytes in the CNS, express and produce MT1/2 to protect enteric neurons [16,33]. In addition, Haton et al. [34] reported that MT1/2-immunopositive signals were detected mainly in the epithelial cells of small intestinal mucosa, which seemed to be stronger in the crypts and at the base of villi compared to the top of villi. In addition, *MT1/2* mRNA and proteins were upregulated by radiation exposure-induced inflammation in the small intestinal mucosa of mice [34]. These finding suggest MT1/2 could be induced in response to inflammation in the ENS as well as the CNS.

### 2.3. Expression of MTs in the Brains of PD Patients

Transcriptome profiling indicated increased expression of *MTs’* genes (*MT1G*, *MT1H*, *MT1L*, *MT1X* and *MT2A*) in the SN and putamen in sporadic PD cases [35]. Michael et al. demonstrated upregulation of MTs’ genes (*MT1E*, *MT1F*, *MT1G*, *MT1H*, *MT1M*, *MT1X* and *MT2A*) and MT1/2 expression in reactive astrocytes in the SN of PD patients [19]. In addition, Glaab et al. performed a statistical meta-analysis of human brain transcriptomics data to investigate potential mechanistic relationships between adult brain aging and PD pathogenesis at the pathway and network level [18]. *MT1G and MT1H* genes were significantly upregulated in both PD and adult brain aging, suggesting MT1 could be a functional molecule with potential applications as combined risk biomarkers to detect aging- and PD-linked oxidative stress [18]. Furthermore, *MTs’* genes (*MT1A*, *MT1E*, *MT1F*, *MT1G*, *MT1M*, *MT1X* and *MT2A*) were upregulated in the prefrontal cortex in PD analyzed by RNA-sequencing study [17]. These findings suggest that MT1/2 could be a novel biomarker for PD diagnosis.

## 3. Protective Functions of MTs against Etiopathogenesis in PD

### 3.1. Metal Chelation

Environmental factors play an important role in the etiology of PD. In particular, exposure to heavy metals, such as Fe, Cu, Zn, Mn, Hg, Pb and Al, has been identified as a potential cause of PD and contributor to disease progression [6]. Epidemiological studies demonstrated an association between metal exposure and PD onset [6]. Exposure to heavy metals is related to the activation of proinflammatory cytokines, resulting in neuronal loss through neuroinflammation. Metals also disrupt redox homeostasis, followed by an increase of free radical production and a decrease of antioxidant levels; thus, metals induce oxidative stress, DNA damage, mitochondrial dysfunction and apoptosis, which can trigger neurodegeneration. In particular, Fe and Cu induce ferroptosis, reported as a new type of Fe-dependent cell death involving the generation of oxidative stress, mitochondrial dysfunction and lipid peroxidation [36]. Furthermore, metals accelerate α-synuclein aggregation [37]. Recently, metal-containing nanoparticles (NPs) have received attention as environmental toxicants implicated in PD [38,39]. Metal-containing NPs, including heavy metals and metal oxides, exist in the environment as components of dust and smoke. Metal-containing NPs, taken into the body via ingestion, inhalation and skin absorption, cross the blood–brain barrier and reach the brain. The mechanisms of neurotoxicity of metal-containing NPs are related to inflammation, oxidative stress, DNA damage and mitochondrial damage as well as to heavy metals [38,40].

**Figure 1:**
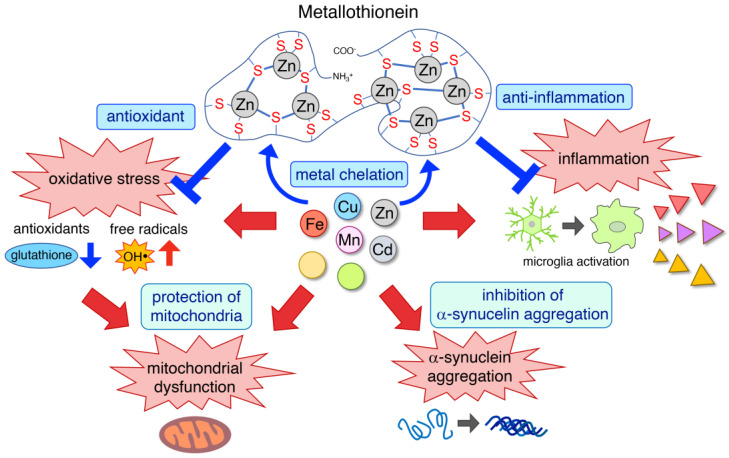
Protective functions of MTs against etiopathogenesis in PD. Heavy metals induce microglial activation, which produces proinflammatory cytokines (indicated by triangles) resulting in neuroinflammation. Metals increase free radicals and decrease antioxidants such as reduced form of glutathione; thus, metals induce oxidative stress and mitochondrial dysfunction. Metals also accelerate α-synuclein aggregation. MTs chelate metals and inhibit metal-induced oxidative stress, inflammation, mitochondrial dysfunction and α-synuclein aggregation. In addition, MTs possess antioxidative properties by scavenging free radicals and exert anti-inflammatory effects by suppression of microglial activation.

MTs are ubiquitous, low molecular weight (500–14,000 Da), cysteine-rich, metal-binding proteins. MTs can bind Cu^+^, Cd^2+^, Zn^2+^ and other 18 different kinds of metals based on their abundant thiol groups, which form metal-thiolate clusters (C-terminal: α-domain, N-terminal: β-domain) and function in metal homeostasis, mainly of Cu and Zn, and detoxification of heavy metals [41]. In the α-domain, four divalent or six monovalent cations can be bound, while in the β-domain, three divalent or six monovalent metal ions are coordinated. The binding affinity varies between metals; the order of displacing 50% of the Zn-MT binding (EC_50_) is Cd (1.33 µM) > Pb (1.46 µM) > Cu (1.93 µM) > Hg (3.93 µM) > Zn (8.06 µM) [42]. Cu has the greatest stability constant, followed by Cd and then Zn. Zn is displaced by Cu^+^, Cd^2+^, Pb^2+^, Ag^+^, Hg^2+^ and Bi^2+^ [43]. As mentioned in Section 2 above, MTs show ubiquitous distribution and have high affinity for metal binding; thus, MTs could capture heavy metals to prevent neurotoxicity. In addition, MTs possess antioxidative, antiapoptotic, and anti-inflammatory properties; accordingly, MTs could be a target for neuroprotection against etiopathogenesis in PD (Figure 1).

#### 3.1.1. Iron

Fe is one of the most common metals and plays a role as a cofactor of proteins involved in various biological functions, such as DNA synthesis, mitochondrial respiration, oxygen transportation and synthesis of neurotransmitters. In the healthy brain, Fe concentrations are higher in the SN and basal ganglia than the other regions [7,44], because Fe functions as a cofactor for tyrosine hydroxylase (TH), a rate-limiting enzyme of DA synthesis [45]. During healthy aging, Fe levels increase in various brain areas, including the SN [46,47]. In PD patients, Fe accumulation is unusually enhanced in the SN and appears to correlate with disease severity [7,46,48,49]. Excess Fe can induce oxidative stress and cell death due to its catalytic function in the production of highly toxic hydroxyl radicals via the Fenton reaction and non-enzymatic oxidation of cytosolic DA to form DA quinones [50]. Fe accumulation also drives the Fe-dependent unique cell death pathway, ferroptosis, which is mediated by Fe-dependent phospholipid peroxidation [51]. Furthermore, free Fe increases α-synuclein aggregation by catalyzing the formation of oligomers [52]. Therefore, DA neurons are vulnerable due to high content of Fe.

Since Fe accumulation in the brain has been linked to PD, metal chelation could be a promising therapeutic approach [53]. Indeed, experimental studies using parkinsonian models have shown that Fe chelation reduces pathological α-synuclein accumulation [54] and oxidative stress [55,56]. A lipid permeable Fe chelator, deferiprone, was reported to improve symptoms in PD patients in clinical trials [57]. However, in a randomized double-blind, placebo-controlled trial, deferiprone could not significantly improve motor Unified Parkinson’s Disease Rating Scale (UPDRS) scores [58]. In the clinical study, relatively short-term administrations of deferiprone for six months showed a tendency to improve motor symptoms, but this could not reach significance. Longer-term clinical trials would be required in the future. MTs can bind to Fe^2+^ with lower affinity than Zn and Cu. We suppose that MTs can inhibit ferroptosis and α-synuclein aggregation by their Fe-binding and anti-oxidative properties [25,59]. Orihuela et al. reported that Zn-MTs complexes could act as electron donors to reduce the Fe^3+^ to Fe^2+^ of ferritin, resulting in Fe^2+^ release from the ferritin [60]. At that time, MT thiolates became fully oxidized to disulfides, which caused Zn^2+^ release. This suggests that the Fe^2+^ release from ferritin by the Zn-MTs complex could occur under an oxidative state. It is still unknown if the release of Fe^2+^ and Zn^2+^ could exert protective or toxic function in the physiological condition.

#### 3.1.2. Copper

Cu functions as an essential cofactor and is required for structural and catalytic proprieties of many important enzymes, such as ceruloplasmin, cytochrome c oxidase, DA hydroxylase, tyrosinase and Cu/Zn-superoxide dismutase (Cu/Zn-SOD; SOD1) [61]. Cu plays a crucial role in essential processes including the synthesis of neurotransmitters, the transformation of energy within mitochondria, antioxidative defenses, and cell signaling [61,62]. MTs can bind Cu with a high affinity. Thus, MTs could act as Cu chaperones to supply the metal to various enzymes.

The concentration of Cu in the brain is highest in the SN [63]. Cu homeostasis is disrupted in PD patients; neuromelanin-bound Cu is decreased in the SN, and the levels of free Cu are increased in the cerebrospinal fluid (CSF) [11] and blood [64,65]. The level of free Cu in the CSF is related to clinical factors such as duration of the disease and score of motor symptoms; therefore, it could be a potential biomarker of PD [66]. Free Cu binds cysteine residues in proteins and inhibits enzymatic activity [67]. Cu also causes oxidative damage by participating in the Fenton and Haber–Weiss reactions, which convert superoxide anions and hydrogen peroxide to hydroxyl radicals [6]. Furthermore, Cu influences Fe content in the brain through ferroxidase ceruloplasmin activity; therefore, decreased protein-bound Cu in the brain could enhance Fe accumulation and consequent oxidative stress. In dopaminergic neurons, Cu promotes DA oxidation, resulting in the production of DA quinones, hydroxyl radicals, superoxide and H_2_O_2_. Furthermore, Cu plays a critical role in the metal-catalyzed oxidative oligomerization of α-synuclein [68]. The binding of α-synuclein to Cu results in not only protein fibrillation but also oxidative stress. It is reported that the complex of α-synuclein-Cu^2+^ can oxidize DA to *o*-quinone in the presence of oxygen. Therefore, chelation of free Cu can lead to dopaminergic neuroprotection in PD. The incubation of these complexes with MT-3 transferred Cu from α-synuclein to MT3, resulting in elimination of oxidative stress and DA oxidation [69]. Due to the Cu-binding ability of MTs, MTs are a potential target for attenuating Cu-induced α-synuclein aggregation [11].

#### 3.1.3. Zinc

Zn plays a crucial role in biochemical pathways and cellular function including DNA and RNA synthesis, DNA replication, transcription, protein synthesis, cellular transport, proliferation, differentiation and the immune system [70]. Zn is required for the structural stability of Zn finger proteins, which are transcriptional factors and regulate signal transduction, cell differentiation or proliferation, cell adhesion and transcription [70]. Zn also exerts antioxidative effect in an indirect manner. Zn maintains the structure at the active site of Cu/Zn-SOD. In addition, Zn induces MT synthesis and maintains an adequate level of MTs [71]. Moreover, Zn also increases reduced glutathione (GSH) synthesis through upregulation of Nrf2 [72]. 

However, Zn exposure has been identified as a risk factor for PD [8]. Recently, it was reported that Zn oxide nanomaterials are environmental toxicants for the onset and development of PD [73]. Post-mortem studies demonstrated excessive Zn depositions in the SN and the striatum of PD patients [49]. Cellular Zn level is tightly controlled by the cooperative function of Zn transporters and MTs. Alteration of intracellular Zn homeostasis is recognized as a key factor in the development of PD. In particular, excess free Zn is cytotoxic and has been reported to be implicated in the pathophysiology of PD. Experimental studies revealed the mechanism of Zn-induced cytotoxicity: oxidative stress through activation of nicotinamide adenine dinucleotide phosphate (NADPH) oxidase and depletion of GSH, which in turn triggers apoptotic neuronal death, activation of microglia and induction of inflammatory cytokines [8]. Chen et al. demonstrated that Zn is also essential for ferroptosis [74]. Furthermore, Zn can accelerate the amyloid fibrillation of α-synuclein [75]. Therefore, Zn chelators are supposed to prevent neurodegeneration in PD.

As mentioned in Section 3.1 above, MTs bind Zn and control cellular Zn concentration. Under oxidative stress, Zn is released from MTs and transferred to Zn-required transcription factors to regulate expression of several genes involved in the growth, proliferation, differentiation and antioxidant pathways [76]. Thus, MTs provide neuroprotection not only as a chelator of free Zn but also by regulating Zn-mediated transcriptional activation.

#### 3.1.4. Manganese

Mn is one of the most important metals in mammals and plays crucial roles in physiological processes, including brain development, neuronal function, antioxidant defense and immune defense [77]. Mn functions as cofactor of multiple enzymes, such as glutamine synthetase (GS), Mn-SOD and pyruvate decarboxylase [78]. Despite its essential physiological roles, Mn also has neurotoxic effects at high concentrations and with prolonged exposure. It is reported that the striatum, globus pallidus, and SN are target sites for Mn accumulation and neurotoxicity [79]. Occupational or environmental exposure to Mn leads to the development of manganism, a neurological condition characterized by motor symptoms, such as bradykinesia, tremor, cock-walk gait and widespread rigidity, similar to PD [80]. Indeed, several studies have investigated the possible association between chronic Mn overexposure and PD [80,81]. A longitudinal cohort study of welding-exposed workers showed that exposure to Mn-containing welding fumes may cause a dose-dependent progression of parkinsonism [81]. Previous studies showed Mn disrupts DA transmission in the striatum [82]. Roth et al. reported that Mn decreases DA uptake and amphetamine-induced DA efflux in DA transporter (DAT)-containing cells by promoting trafficking of cell surface DAT into intracellular compartments [83]. The cellular mechanism of Mn-induced neurotoxicity consists of oxidative stress, mitochondrial dysfunction and neuroinflammation [6]. In addition, recent studies demonstrated that Mn exposure affects α-synuclein synthesis, aggregation and cytotoxicity [84,85]. Harischandra et al. showed that Mn promoted the aggregation and prion-like cell-to-cell exosomal transmission of α-synuclein [84].

Relationship between Mn and MTs are not well documented. MTs are thought to lack Mn’s binding property, but MTs are inducible in response to Mn exposure. Erikson et al. reported that Mn exposure decreased *MT* mRNA levels in astrocytes [86]. Taken together, it is suggested MTs’ induction may occur in response to oxidative stress or inflammation by Mn exposure.

#### 3.1.5. Cadmium

A toxic, non-essential transition metal, Cd has a long biological half-life due to its low rate of excretion from the body. Thus, prolonged exposure to Cd causes its accumulation in tissues and exerts toxicity. Chronic Cd exposure increases blood–brain barrier (BBB) permeability by decreasing the cellular antioxidant defenses. As a consequence, Cd can reach the brain more easily [87,88]. The main mechanism of the neurotoxic action of Cd is thought to be oxidative stress. Cd produces reactive oxygen species (ROS) via indirect mechanisms by weakening antioxidative enzymes, such as catalase, glutathione-S-transferase (GST), glutathione peroxidase (GPx) and SOD [89]. In addition, mitochondrial dysfunction, GSH depletion, lipid peroxidation and endoplasmic reticulum (ER) stress are involved in molecular pathway of Cd neurotoxicity [87,90].

Environmental and occupational exposures to Cd increase the risk of PD. Okuda et al. reported a case in a welder who developed parkinsonism after acute Cd exposure [91]. Cd neurotoxicity induces motor dysfunctions because Cd affects DA receptors. Gupta et al. reported Cd exposure decreases expression of DA-D2 receptors in the striatum of rat brains [92]. Moreover, they demonstrated that the decrease in DA-D2 receptors may be due to direct binding of Cd at the competitive site of DA on DA-D2 receptors [92]. Yu et al. explored alteration of gene expression after Cd exposure in mouse embryonic fibroblast cells [93]. They identified gene expression changes in the ubiquitin-proteasome system, in antioxidant and phase II enzymes, and in genes involved in cell cycle regulation pathways. Furthermore, pathway analysis revealed significant alterations in genes implicated in PD pathogenesis [93].

It is well known that Cd exposure induces MTs synthesis via MTF-1 transcriptional activation [94]. Experimental studies demonstrated protective effects of MTs against Cd toxicity, but most of them were investigated in the peripheral tissues, liver and kidney [95]. Still, we can suppose that MTs also could exert neuroprotective effects in the CNS due to not only Cd absorption but also strong antioxidative action.

#### 3.1.6. Other Metals

Se, an essential trace element, plays a critical role in the CNS via synthesized selenoproteins, which include various antioxidant enzymes such as GPx, thioredoxin reductase and thioredoxin-glutathione reductase [96]. A deficiency of Se contributes to the pathogenesis of neurodegenerative diseases such as PD. In addition, Se overexposure leads to neurotoxicity. It has been demonstrated that Se levels in the CSF of PD patients were significantly higher than those in controls [97,98]. In experimental studies, Se treatment exhibits neuroprotective effects in parkinsonian models [99,100,101]. Ellwanger et al. reported that Se administration reduced DNA damage and bradykinesia in parkinsonian rats injected with paraquat, which is a pesticide associated with increased risk for developing PD [99]. Sun et al. demonstrated that administration of sodium selenite increased GPx activity and provided neuroprotective effects in 1-methyl 4-phenyl 1,2,3,6-tetrahydropyridine (MPTP)-injected parkinsonian mice [100]. It is still unknown whether MTs can function in the maintenance of Se; however, it is reported that sodium selenite induces MTs in mice. Furthermore, He et al. demonstrated Se-MT complex formation by MALDI–TOF-MS and X-ray absorption spectrometry [102]. Mg is essential for various biological processes such as enzymatic reactions, ion channel functions and cellular signaling. Mitochondria function as a major cellular Mg^2+^ pool and regulate intracellular Mg^2+^ homeostasis [103]. Rajput et al. measured Mg concentration in the scalp hair and blood of PD patients; Mg concentration was higher in the scalp hair and lower in the blood of PD patients at early to severe stages compared to control group [104]. Shen et al. demonstrated that treatment with magnesium-L-threonate attenuated motor deficits and dopaminergic neurotoxicity in MPTP-injected PD model mice [105]. MTs can bind Mg^2+^, but its binding affinity is low [42]. The role of MTs in homeostasis and regulation of Mg is not fully documented. Kotani et al. reported that Mg deficiency increased MT proteins with upregulation of *MT1* and *MT2* mRNA in rat liver [106], suggesting the interaction of MTs and Mg.

### 3.2. Antioxidant

The etiology of PD and the mechanisms of neurodegeneration remain not fully elucidated. Mitochondrial dysfunction, neuroinflammation and environmental factors are considered as key pathological events in both familial and sporadic PD. Oxidative stress, including the generation of ROS and reactive nitrogen species (RNS), is believed to be the common mechanism leading to neuronal death in PD. In particular, oxidative stress is thought to be an important pathogenic determinant in dopaminergic neurodegeneration. DA is stable in the synaptic vesicle; however, excess cytosolic DA outside of the synaptic vesicle is easily metabolized via monoamine oxidase (MAO) or by autooxidation to produce cytotoxic ROS and subsequent neuromelanin formation [107]. H_2_O_2_ is produced in the metabolic process of DA by MAO, and superoxide and reactive quinones are generated in the non-enzymatical and spontaneous autooxidation of DA [108]. Generated superoxide reacts with nitric oxide radicals to consequently generate peroxynitrite of the RNS. As mentioned in Section 3.1.1 and Section 3.1.2 above, dopaminergic neurons are rich in metals; therefore, the most cytotoxic hydroxyl radicals are likely to be generated due to the reaction between H_2_O_2_ and metals, especially Fe. In contrast to the general oxidative stress induced by ROS or RNS, the pathogenicity of DA quinone formation has received attention as dopaminergic neuron-specific oxidative stress [109,110,111]. DA quinones are generated by not only autooxidation but also by enzymatic oxidation by cyclooxygenase (COX) as prostaglandin H synthase, lipoxygenase, tyrosinase and xanthine oxidase [108,112,113,114,115]. These quinones are easily oxidized to the cyclized aminochromes, DA-chrome and DOPA-chrome, and then polymerized to form melanin. The DA quinones interact with various bioactive molecules. The DA quinones covalently conjugate with the cysteine residues of functional proteins to subsequently form quinoproteins and inhibit their functions. DAT; parkin (PARK2), an E3 ubiquitin ligase; and TH are recognized as targets of DA quinones [116,117,118,119]. Furthermore, DA quinones cause mitochondrial dysfunction, inflammation and proteasome impairment [120,121,122]. α-synuclein is also one of the target proteins for DA quinones [123]. Conway et al. reported that DA quinones react with α-synuclein to form a quinoprotein DA quinone-α-synuclein adduct, which inhibits the conversion of toxic protofibrils to fibrils, causing accumulation of the α-synuclein protofibril [123]. These findings suggest that DA quinone formation is a common toxic factor involved in dopaminergic neurodegenerative pathways. 

MTs are well known as powerful antioxidants which can scavenge a variety of radicals including superoxide and hydroxyl radicals. In particular, the rate constant for the reaction of MTs with hydroxyl radicals is extremely high; that is, about 300 times more than GSH, which is recognized as the most potent intrinsic antioxidant [124]. The cysteine residues of MTs are thought to be important for radical scavenging. The hydroxyl radicals target primarily cysteine residues of proteins. It is also reported that MTs attenuate peroxynitrite-induced oxidative stress in PD models [125]. Another mechanism of the antioxidant function of MTs is metal chelating. As mentioned in Section 3.1 above, metals generate free radicals and oxidative stress. MTs act as strong metal chelators to inhibit radical generating reactions. In particular, MT1/2 are important for dopaminergic neuroprotection. In addition to the scavenging ability of general oxidative stress, MTs possess a quenching property of DA quinones by binding through cysteine residues. We reported that MT1 directly quenched DA semiquinones in vitro [126]. In addition, our previous studies demonstrated that excess DA upregulated MT1/2 levels specifically in astrocytes, and that MTs secreted from astrocytes protected dopaminergic neurons against DA quinone toxicity in parkinsonian models [21,126]. Gauthier et al. reported that MTs form covalent arylation products with DA and 6-hydroxydopamine (6-OHDA) and protect dopaminergic neurons [127]. MTs also inhibit Charnoly body formation, which is a pleomorphic, multi-lamellar, electron-dense stack of degenerated membranes formed in the most vulnerable cells due to compromised mitochondrial bioenergetics in severe malnutrition and free radical overproduction by their antioxidative actions [128,129,130]. Moreover, MTs and GSH cooperate to maintain the redox state. When MTs react with ROS or oxidized glutathione (GSSG), MTs release Zn and form MT disulfides at the sulfhydryl groups of its cysteine residues, which can be reduced by GSH in the presence of selenium to consequently reconstitute Zn-binding MTs [131]. In other words, MTs and GSH reduce each other via their cysteine residues and maintain antioxidative machinery.

### 3.3. Anti-Inflammation

Chronic neuroinflammation is consistently associated with the pathophysiology of PD. Activated glial cells, mainly microglia, are major players in neuroinflammation. In response to stimuli, microglia release pro-inflammatory cytokines, such as IL-1β, IL-6, IL-8, IL-12, IL-18, TNF-α, interferon-γ (IFN-γ) and prostaglandins, which can subsequently enhance microglial activation through autocrine signaling [132]. Indeed, microglial activation and increase in cytokines, IL-1β, IL-2, IL-6 and TNF-α, were observed in the SN of PD patients [132,133]. In addition, IL-1β levels in plasma have a positive correlation with the Hoehn and Yahr staging scale and UPDRS part III scores [134]. Moreover, microglia become activated in response to neuronal damage; thus, the activation is accelerated by positive feedback from degenerating neurons. Therefore, inhibiting the repetitive cycle of neurotoxic microglial activation is important for neuroprotection. It is especially crucial to PD because dopaminergic neurons in the SN pars compacta (SNpc) are particularly susceptible to microglia-mediated neurotoxicity due to the high densities of microglia present [135]. Experimental studies demonstrate that DA neurons are more vulnerable to inflammatory stimuli than other cell types. Castaño et al. reported that the intranigral injection of LPS, an endotoxin from Gram-negative bacteria, induces dopaminergic neuronal loss [136]. In addition, Aloe et al. demonstrated that transgenic mice expressing TNF-α specifically in the brain induced dopaminergic neurotoxicity [137].

MTs exert anti-inflammatory effects in the brain; the main mechanism is the reduction of IL-1, IL-6, IL-12 and TNF-α [10,138]. Penkowa et al. reported that *MT1* overexpression reduced the level of IL-1, IL-6, IL-12, TNF-α and matrix metalloproteinases (MMP-3, MMP-9) and increased anti-inflammatory IL-10 in the hippocampus of transgenic mice relative to wild-type mice [10]. Various studies demonstrated that IL-1, IL-6 and TNF-α induce *MTs’* expression via STAT signaling [31,139,140,141]. It is suggested that this cytokine-induced MT upregulation would be a protective reaction against inflammation. The mechanisms for reduction of inflammatory cytokines by MTs are thought to be the suppression of microglial activation and Zn-mediated actions. Giralt et al. demonstrated that *MT1*-overexpressing mice exhibit less microglial activation and reduction in proinflammatory cytokines IL-1β, IL-6 and TNF-α following traumatic brain injury [142]. In contrast, it is reported that *MT* knockout increased microglial activation following mouse brain injury [143]. These findings indicate MTs can inhibit microglial activation. As mentioned in Section 3.1.3 above, MTs regulate Zn availability by supplying Zn and chelating excess Zn. It is well known that Zn inhibits inflammatory responses [144]. Hongxia et al. reported that Zn inhibited inflammatory responses mediated by microglia via upregulation of Zn finger protein A20 (TNF-α induced protein-3, TNFAIP3), which is known as TNF-induced and NF-κB transcription-dependent inflammatory inhibitor [145]. Conversely, Zn also exhibits neurotoxicity. Therefore, controlling the appropriate Zn concentration by MTs is extremely important.

### 3.4. Protection of Mitochondria

Evidence indicates that mitochondrial dysfunction is closely associated with pathogenesis of PD; that is, a feature common to sporadic and familial PD. Dopaminergic neurons in the SNpc require higher ATP than other cells, because a single DA neuron has a large number of projections into the striatum [146]. Indeed, nigral DA neurons show significantly higher density of axonal mitochondria and higher levels of peroxisome proliferator-activated receptor-γ coactivator 1α (PGC-1α), a master regulator of mitochondrial biogenesis, and basal oxygen consumption rates [146,147,148]. Therefore, mitochondrial dysfunction is a key contributor to vulnerability of dopaminergic neurons. Mitophagy, a selective degradation of impaired mitochondria, is an essential process in the maintenance of the health of mitochondria. Increasing evidence has shown that neurodegeneration is accompanied by abnormal mitophagy. Parkin and PTEN-induced putative kinase 1 (PINK1), whose mutations are associated with early-onset autosomal recessive PD, cooperate to degrade impaired mitochondria via mitophagy [149,150,151].

A few studies have reported on the mitochondrial protective effects of MTs. The main functions of MTs, the maintaining of the metal homeostasis and the redox balance, are thought to directly link to the mitochondrial functions. Mitochondria require Cu for the function of the respiratory complex IV, cytochrome c oxidase, and the antioxidant enzyme Cu/Zn-SOD [152]. Cu plays an important role in mitochondrial function and signaling involving bioenergetics, dynamics and mitophagy [152]. Because MTs act as Cu chaperones, MTs could play an important role in the maintenance or recovery of mitochondrial activity. Saini et al. demonstrated that MT upregulation by MTF-1 overproduction rescued mitochondria in a parkin-mutant fly [153]. Kang et al. reported that delivery of antioxidant MTs to mitochondria by using cell-penetrating artificial mitochondria-targeting peptide-conjugated human MT1A (CAMP-hMT1A) increased mitochondrial function and restored TH expression and mitochondrial activity in cultured cells [154]. Furthermore, stereotaxic injection of CAMP-hMT1A into the SN of parkinsonian model mice inhibited dopaminergic neurodegeneration and rescued movement impairment [154].

Cytosolic Zn^2+^ can enter mitochondria and induce loss of mitochondrial membrane potential, mitochondrial swelling and ROS generation and reduce cellular ATP levels [155]. In addition, mitochondria act as the major Fe recipients [156]. Post-mortem examinations revealed that Fe accumulation is predominantly present in the mitochondria. Fe accumulation in the mitochondria leads to bioenergetic disturbances and the generation of ROS [157]. Prasuhn et al. demonstrated that brain Fe deposition was highly predictive of mitochondrial impairment in idiopathic PD patients, suggesting the significance of chelating agents in treatments for PD patients [158]. Thus, MTs could inhibit mitochondrial dysfunction by chelating excess metals.

### 3.5. Inhibition of α-Synuclein Aggregation

The pathological hallmark of PD is the presence of Lewy bodies and neurites, which contain α-synuclein aggregates. Braak et al. [159] introduced a pathological staging scheme for PD within the CNS in which α-synuclein aggregates appeared first in the olfactory bulb and the dorsal motor nucleus of the vagus (DMV) in the brainstem, and then spread progressively through the SN, eventually leading to motor dysfunction, to reach the cerebral cortex. Importantly, the spread pattern of PD pathology correlates with the progression of clinical symptoms. In addition, several reports have demonstrated that PD pathology is also detected within the peripheral nervous system (PNS): the intestinal myenteric plexus, gastric mucosa, cardiac sympathetic nerve and skin nerve. Constipation is a typical prodromal non-motor symptom in PD, which precedes motor symptoms by 10–20 years [160]. Therefore, it has been hypothesized that PD pathology propagates from the ENS to the CNS via the vagal nerve [4].

Aggregates of α-synuclein are released from neuronal cells and undergo cell-to-cell propagation in a prion-like fashion. The α-synuclein aggregates taken up by neighboring cells facilitate interactions with endogenous α-synuclein monomers and other cytosolic proteins and further promote α-synuclein aggregation and propagation [161,162,163]. In the recipient cells, the α-synuclein aggregates cause oxidative stress, mitochondrial impairment, ER stress, disruption of proteasomal protein clearance and synaptic impairment [164]. In addition, neuron-to-glia transmission of α-synuclein aggregates, followed by its accumulation and deposition in glial cells, can cause neuroinflammation and contribute to neurodegeneration in PD.

Heavy metals, such as Fe, Cu and Mn, directly accelerate α-synuclein fibril formation by inducing conformational changes [165]. Cu^2+^ is the most effective metal ion at enhancing α-synuclein oligomerization [166]. The binding of α-synuclein to Cu results in not only protein fibrillation but also oxidative stress. The complex of α-synuclein-Cu^2+^, the major component of intracellular Lewy body inclusions in nigral dopaminergic neurons, can catalyze toxic reactions such as ROS production through catalytic Cu^+^/Cu^2+^ redox-cycling, amino acid side-chain oxidation and oligomer formation. The α-synuclein-Cu^2+^ complex also oxidizes DA to o-quinone [69,167,168]. Furthermore, DA quinone reacts with α-synuclein to form covalent adducts, which prolongs the lifetime of pathogenic α-synuclein protofibrils [123]. As mentioned in Section 3.1.2 above, MTs can bind Cu with a high affinity. Additionally, MTs directly and strongly quench DA quinones. Therefore, MTs could be a potential target for attenuating Cu-induced α-synuclein aggregation and neurodegeneration. Experimental studies revealed that MTs inhibit Cu-induced α-synuclein aggregation [69,169]. Meloni et al. demonstrated that MT-3 (Zn_7_MT-3) removed Cu^2+^ from the complex of α-synuclein-Cu^2+^ and quenched the α-synuclein-Cu^2+^-induced DA oxidation and ascorbate-driven Cu-catalyzed hydroxyl radicals [69]. Calvo et al. also reported that Zn_7_MT-3 could efficiently target soluble and membrane-bound α-synuclein-Cu^2+^ complexes through Cu^2+^ removal [170]. The Zn_7_MT-3 completely abolished DA quinone generated by membrane-bound α-synuclein-Cu^2+^ complexes. In addition, McLeary et al. reported that the synthetic glucocorticoid analogue, dexamethasone, upregulated *MT* gene expression and reduced Cu-dependent α-synuclein intracellular aggregation [169].

Intracellular Fe and α-synuclein may act in a vicious reaction cycle of toxicity that contributes to the vulnerability of DA neurons. A variety of post-translational modifications, including oxidation and nitration by α-synuclein, are accelerated by excess Fe and Fe-induced oxidative stress [46]. Fe directly binds to the C-terminus of α-synuclein [171]. This ability of α-synuclein to bind Fe promotes its aggregation into fibrils by inducing conformational changes, facilitating α-synuclein oligomerization [165]. In addition, occupational exposure to Mn containing welding fumes increases risk of parkinsonism [172]. Experimental studies demonstrated that Mn exposure induced oligomeric α-synuclein secretion in exosomes and accelerated cell-to-cell transmission of α-synuclein oligomers, resulting in neuronal death by neuroinflammation [84]. MTs, particularly MT1A/2A, are sensitively and dramatically induced in response to metal exposure. Astrocytes produce MT1A/2A and secrete extracellularly. The extracellular MT1A/2A are taken up by neurons via LRP1 and megalin. Thus, MTs can comprehensively prevent intra- and extracellular metal-induced α-synuclein aggregation and prion-like cell-to-cell propagation leading to neurodegeneration.

## 4. Neuroprotective Approaches Targeting MTs in PD

### 4.1. Neuroprotection against Nigral Dopaminergic Neurodegeneration

**Figure 2:**
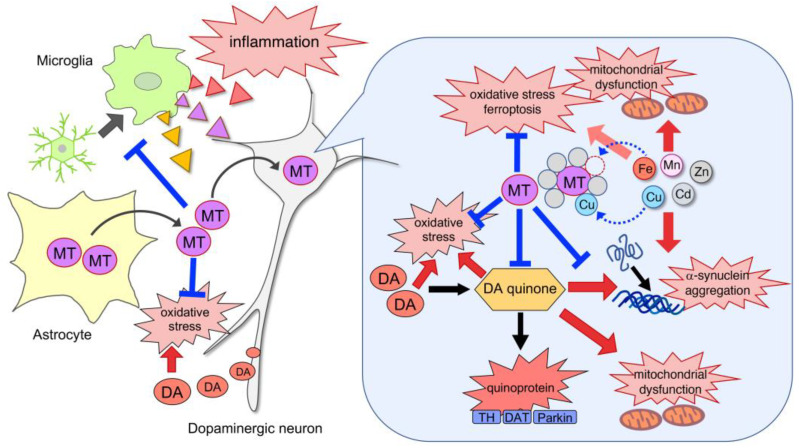
Neuroprotective functions of MTs against dopaminergic neurotoxicity. MT1A/2A are produced in astrocytes and secreted extracellularly. In the extracellular space, MTs ameliorate oxidative stress and microglial inflammatory actions by secretion of inflammatory cytokines (triangles). Extracellular MTs are taken up by dopaminergic neurons and the MTs exert neuroprotective effects. Dopaminergic neurons are rich in DA and metals. Cytosolic free DA produces free radicals and DA quinones, dopaminergic neuron-specific oxidative stress. DA quinones interact with TH, DAT and Parkin to form quinoproteins and inhibit their functions. DA quinones also cause mitochondrial dysfunction and α-synuclein aggregation. Metals, especially Fe and Cu, induce oxidative stress, resulting in ferroptosis, mitochondrial dysfunction and α-synuclein aggregation. MTs reduce directly oxidative stress induced by DA and metals. In addition, MT1 quenches DA quinones and inhibit DA quinone toxicity. Furthermore, MTs chelate metals (circles) and prevent metal-induced neurodegeneration.

MTs possess multiple functions which attenuate various neurotoxic factors associated with PD; therefore, upregulation of MTs could be a therapeutic strategy for neuroprotection in PD (Figure 2). 

Our previous studies demonstrated that serotonin 1A (5-HT1A) agonist, (R)-(+)-8-hydroxy-2-(di-n-propylamino) tetralin hydrobromide (8-OH-DPAT), promoted astrocyte proliferation and the upregulation of MT1/2 via Nrf2 nuclear translocation. The conditioned media from 8-OH-DPAT-treated astrocytes contained MT1 and protected dopaminergic neurons against oxidative stress. These results indicate that the 5-HT1A agonist upregulates MT1/2 expression in astrocytes and promotes MTs’ secretion into extracellular space, resulting in dopaminergic neuroprotection. Administration with 8-OH-DPAT inhibited dopaminergic neurodegeneration in 6-OHDA-injected parkinsonian mice [15]. In addition, our recent experiments showed that the antiparkinsonian drug rotigotine, which is a DA agonist and a 5-HT1A partial agonist, induced MT1/2 production and secretion in/from astrocytes, and ameliorated dopaminergic neurodegeneration in parkinsonian models [13]. We confirmed the upregulation of MT1/2 in astrocytes and neuroprotective effects of rotigotine via 5-HT1A receptors. Furthermore, we confirmed neuroprotective effects of 5-HT1A agonists using mirtazapine, a noradrenergic and specific serotonergic antidepressant (NaSSA). Mirtazapine, which indirectly stimulates 5-HT1A receptors, also upregulated MT1/2 in astrocytes via 5-HT1A receptors and prevented dopaminergic neurodegeneration [14]. Taken together, MT1/2 upregulation by stimulation of 5-HT1A receptors on astrocytes could provide a promising therapeutic strategy for neuroprotection in the treatment of PD.

Other drugs have also been reported to have neuroprotective effects targeting MTs. Choudhury et al. reported that anti-parkinsonian drug zonisamide upregulated antioxidative proteins, such as MT2A, Cu/Zn-SOD and Mn-SOD, and neurotrophic factors in astrocytes and protected nigral DA neurons in 6-OHDA-injected parkinsonian rats [173]. Ono et al. reported that an ergot-derived DA agonist, pergolide, recovered *MT1* mRNA and upregulated *MT3* mRNA expression in the striatum of MPTP-injected parkinsonian mice [174].

Epidemiological studies indicate that coffee consumption decreases the risk of PD. It is also reported that pesticide exposure, particularly rotenone and paraquat, increases the risk of PD [175]. We examined the neuroprotective effects of caffeic acid (CA) and chlorogenic acid (CGA), which are coffee components and antioxidants, against dopaminergic neurotoxicity in rotenone-exposed parkinsonian models [16]. CA or CGA upregulated MT1/2 production in striatal astrocytes and prevented rotenone-induced dopaminergic neurodegeneration both in primary cultured cells and parkinsonian mice [16]. Furthermore, recent studies demonstrated that extracts of *Eucommia ulmoides* (EU), a traditional herbal medicine, recovered MT1/2 production in astrocytes and ameliorated dopaminergic neurodegeneration and α-synuclein accumulation in the SNpc [12]. EU extracts also prevented rotenone-induced microglial activation [12].

### 4.2. Neuroprotection in the Intestinal Myenteric Plexus

Enteric glial cells (EGCs) in the ENS are a counterpart of astrocytes in the CNS. EGCs express GFAP and represent morphological features of astrocytes [176,177]. EGCs are reported to play an important role in neuroprotection in the ENS. Abdo et al. demonstrated neuroprotective effects of EGCs against oxidative stress by GSH production [178]. Our previous studies demonstrated that GFAP-positive EGCs express MT1/2 in primary cultured cells from intestines of SD rat embryos [16] and colonic myenteric plexus of mice [33]. Neuroprotective effects of MT1/2 in the ENS are indicated by the previous studies using rotenone-injected parkinsonian mice [16,33]; subcutaneous administration of rotenone caused loss of enteric neurons [16,33,179] and decreased gastrointestinal motility [179]. The neuronal loss of the myenteric plexus was aggravated in MT1/2-knockout mice compared to wild-type mice [33]. Upregulation of MT1/2 in EGCs by treatment with CA and CGA could provide neuroprotection in the ENS in rotenone-treated PD models [16]. EU extracts, which can affect MT expression in astrocytes, also exerted protective effects against intestinal neuronal loss in PD model mice [12]. In addition, Foligné et al. demonstrated that supplementation with Zn induced the expression of *MT1/2*-encoding genes in the ileum and colon of mice and prevented gut inflammation [180]. Recently, it was demonstrated that ATH434, a small molecule, orally bioavailable, moderate-affinity Fe chelator, can reverse some of the gastrointestinal deficits and enteric neuropathy in A53T α-synuclein transgenic mice, suggesting that metal chelation could be neuroprotective for enteric neurons [181].

**Figure 3:**
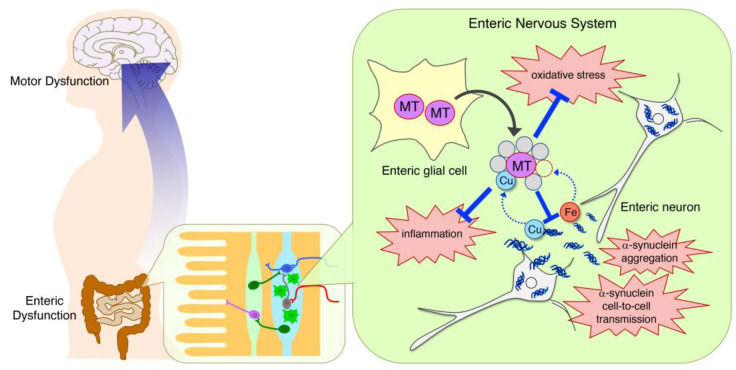
Neuroprotective functions of MTs in the ENS. MT1/2 are expressed in enteric glial cells and secreted extracellularly. In the extracellular space, MTs ameliorate oxidative stress and inflammation. In PD, aggregated α-synuclein is released from neuronal cells and undergoes cell-to-cell propagation in a prion-like fashion. The α-synuclein aggregates are taken up by neighboring cells and further promote α-synuclein aggregation and propagation. MTs chelate metals and prevent metal-induced α-synuclein aggregation and cell-to-cell transmission. Therefore, MTs’ upregulation in the ENS could be a potent therapeutic strategy to inhibit the initial enteric pathogenesis and progression of PD.

Enteric manifestations of PD often precede motor dysfunction by 10–20 years. Accumulating evidence indicates that the ENS is involved in the pathological progression of PD towards the CNS [182]. Therefore, it is desirable to develop approaches that can inhibit enteric neurodegeneration. MTs’ upregulation in the ENS could be a potent therapeutic strategy to inhibit the initial enteric pathogenesis and progression of PD (Figure 3).

## 5. Conclusions

MTs possess various neuroprotective properties, such as antioxidative, antiapoptotic and anti-inflammatory effects, which are effective in a wide range of neurodegenerative diseases. These functions are based on the direct quenching of free radicals and DA quinones, metal chelation and inhibition of microglial activation. Furthermore, MTs prevent α-synuclein aggregation by binding to metals with a high affinity. MTs, particularly MT1A/2A, are sensitively and dramatically induced in response to various stimuli. In the CNS, astrocytes produce MT1A/2A and secrete extracellularly. The extracellular MT1A/2A exert neuroprotective function in the extracellular space; in addition, MTs are taken up by neurons via LRP1 and megalin and prevent intraneuronal pathological pathways. Thus, MTs can comprehensively prevent intra- and extracellular metal-induced α-synuclein aggregation and prion-like cell-to-cell propagation leading to neurodegeneration. Moreover, MT1/2 are expressed by peripheral enteric glia and protect enteric neurons. Currently, it has been hypothesized that PD pathology propagates from the ENS to the CNS via the vagal nerve. Therefore, upregulation of MTs could be a therapeutic approach to prevent the primary degenerative process of PD.A deeper understanding of MTs can help design new therapies that provide neuroprotection against neurodegenerative diseases.
